# 32097 Title V Medical Sciences Campus Project (TVMSC) : Clinical and Translational Research (CTR) with an Interdisciplinary/Entrepreneurship (IE) approach for Students and Faculty (UgS, UgF) from Undergraduate Programs (UgP) in Puerto Rico: an initiative for an early jumpstart in CTR and Scientific Entrepreneurship (SE) in a virtual scenario 2020-25.

**DOI:** 10.1017/cts.2021.554

**Published:** 2021-03-30

**Authors:** Margarita Irizarry-Ramírez, Rubén García García, Edgardo Rosado Santiago, Lizbelle De Jesus-Ojeda, Efrain Flores Rivera, Juan C. Soto Santiago, Maribel Campos n Rivera

**Affiliations:** University of Puerto Rico-Medical Sciences Campus

## Abstract

ABSTRACT IMPACT: This presentation highlights an integrated curriculum in CTR and a scientific entrepreneurship approach to entice and support students and faculty in HP programs into CTR and SE thus expanding the pool of new minority CTR researchers. OBJECTIVES/GOALS: To present the TVMSC as a hub for trainings, mentoring programs, courses, entrepreneurship and support activities for health professionals(HP) and HP students :graduate (GS) and UgS and UgF. Responding to the need for CTR minority researchers, in a virtual setting due to COVID-19 crisis. METHODS/STUDY POPULATION: TVMSC will offer an educational program based in the Center for Research,Entrepreneurship and Scientific Collaboration (CRESCO) with on line courses and workshops in CTR and SE, for HP and students and a continued education curriculum for HP and clinician scientists toward a certification in CTR. Two hands-on experiences: a) a Pilot project program(PiP) with teams composed of an F, that previously completed training cycles and a research experience from a previous project in CTR as PI, with a research mentor and students or an established researcher as a PI with UgS and UgF, and b) participation in a SE team which will engage in training and submission of an SE project proposal. RESULTS/ANTICIPATED RESULTS: By the end of the five-year period the project will have had 200 UgS, 200 GS and 200 F that received online assistance in CTR skills, statistics and SE; 48 UgS and 48 GS with the skills in SEFL. In curricular development the project expects to have 6 online tutorials created, one FLSE online course and 18 modules in CTR content areas available for continued education of HP. Certifications in CTR will be completed by 160F/HPs. The expected participation in CTR on-hands experiences is 32 F, 64 students and 32 established researchers. PiP teams will publish at least 8 scientific papers and SEFL teams will submit at least 5 SE project proposals and 100% increase in CRESCO web based resources DISCUSSION/SIGNIFICANCE OF FINDINGS: This Project and its expected results will provide students and faculty members island-wide with the knowledge, skills and experiences in CTR with IE approach to foster the expansion of a cadre of Hispanic minority CTR researchers in direct benefit of the health of the people of Puerto Rico.

